# Point-of-care laboratory testing in primary care: utilization, limitations and perspectives of general practitioners in Germany

**DOI:** 10.1186/s12875-023-02054-0

**Published:** 2023-04-11

**Authors:** Anni Matthes, Florian Wolf, Guido Schmiemann, Ildikó Gágyor, Jutta Bleidorn, Robby Markwart

**Affiliations:** 1 Institute of General Practice and Family Medicine, Jena University Hospital, Friedrich Schiller University, Jena, Germany; 2 InfectoGnostics Research Campus Jena, Jena, Germany; 3 Institute for Public Health and Nursing Sciences, Department for Health Services Research, Bremen University, Bremen, Germany; 4 Department of General Practice, University Hospital Wuerzburg, Würzburg, Germany

**Keywords:** Point-of-care-tests, POCTs, Rapid tests, primary care, GP practice, Germany, Utilization, Diagnostic procedures, Survey

## Abstract

**Background:**

Due to their fast turnaround time and user-friendliness, point-of-care tests (POCTs) possess a great potential in primary care. The purpose of the study was to assess general practitioners’ (GPs) perspectives on POCT use in German primary care, including utilization, limitations and requirements.

**Methods:**

We conducted a cross-sectional survey study among GPs in Germany (federal states of Thuringia, Bremen and Bavaria (Lower Franconia), study period: 04/22–06/2022).

**Results:**

From 2,014 GPs reached, 292 participated in our study (response rate: 14.5%). The median number of POCTs used per GP was 7.0 (IQR: 5.0–8.0). Six POCTs are used by the majority of surveyed GPs (> 50%): urine dipstick tests (99%), glucose (urine [91%] and plasma [69%]), SARS-CoV-2 (80%), urine microalbumin (77%), troponin I/T (74%) and prothrombin time / international normalized ratio (65%). The number of utilized POCTs did not differ between GP practice type (*p* = 0.307) and population size of GP practice location (*p* = 0.099). The great majority of participating German GPs (93%) rated POCTs as useful diagnostic tools in the GP practice. GPs ranked immediate decisions on patient management and the increase in diagnostic certainty as the most important reasons for performing POCTs. The most frequently reported limitations of POCT use in the GP practice were economic aspects (high costs and inadequate reimbursement), concerns regarding diagnostic accuracy, and difficulties to integrate POCT-testing into practice routines (e.g. time and personnel expenses).

**Conclusion:**

Although participating German GPs generally perceive POCTs as useful diagnostic tools and numerous POCTs are available, several test-related and contextual factors contribute to the relatively low utilization of POCTs in primary care.

**Supplementary Information:**

The online version contains supplementary material available at 10.1186/s12875-023-02054-0.

## Introduction

Point-of-care-tests (POCTs), also referred to as rapid tests, are laboratory procedures that are performed in the close proximity of patients and are characterized by their fast turnaround time as they typically yield results within 30 min [1]. POCTs support clinical-decision making during or very close to the time of consultation, which is particularly important in primary care where clinical decisions are made during the physician–patient-encounter [2].

In the past years, the number of POCTs available for general practitioners (GPs) has steadily increased [3, 4]. Numerous studies on POCTs in outpatient care were performed, predominantly investigating the diagnostic accuracy of POCTs [5, 6]. However, as shown by a regional survey, GPs in Germany only utilize a relatively small number of POCTs in routine healthcare [7]. These tests include urine dipstick tests, glucose, troponin, microalbumin and D-dimer. The limited use of POCTs in German primary care might be explained by several factors, such as reimbursement, feasibility and knowledge. Moreover, international studies indicate that physicians perceive only a small number of POCTs as valuable in clinical practice [8–10]. In addition to medical care in GP practices, German GPs also often provide medical care in home visits, for nursing home residents and in out-of-hours emergency services. The perspectives of German GPs for POCT use in these outpatient care settings have not been studied yet. Integrating the perspectives and needs of physicians in the development of POCTs is pivotal for a successful implementation of novel POCTs for the use in the general practice. We therefore conducted a cross-sectional survey on POCT utilization, limitations and attitudes of GPs in Germany. Moreover, we investigated whether various factors, such as GP work experience, GP practice type and time to receive test results from external laboratories, are associated with POCT utilization.

## Methods

### Study design

We conducted a cross-sectional questionnaire-based survey study among outpatient general practitioners in Germany. In addition to published research, results and experiences from our qualitative research approaches (focus group discussions and individual interviews) with GPs and POCT manufacturers were incorporated in the design of this study. In total, 2,052 GPs from three German federal states received the questionnaire via mail and were asked to participate in our study (Thuringia: *n* = 1,378, Bremen: *n* = 456, Bavaria [Lower Franconia]: *n* = 218, study period: April 14^th^ – June 30^th^ 2022). Due to incorrect address data, 38 letters were returned. Other than working as a GP, no other inclusion or exclusion criteria were applied. Contact information from GPs were derived from existing contact lists at our institutes. Each potential participant was contacted once. Questionnaires were sent back in a prepaid reply envelope. The study complies with the declaration of Helsinki and ethical approval was obtained from the Institutional Research Ethics Board of the Jena University Hospital (Registration No.: 2022–2594-Bef.). The participants received no financial incentives for participating in the survey but we applied several strategies [11] to increase the response rate, including pre-notification (newsletters, homepages, etc.), use of stamped return envelopes, mentioning university sponsorship and unconditional incentives (summary of the survey results and an overview of reimbursable laboratory tests for GP practices listed in the German doctor’s fee scale). This study is part of the project POCT-ambulant (POCT-ambulatory) within the InfectoGnostics Research Campus Jena.

### Questionnaire

The written questionnaire included twelve questions on POCT utilization and limitations as well as perspectives and attitudes of GPs towards POCT use in the general practice. In addition, participants were asked to provide individual characteristics, including gender, work experience, type of practice, employment status, population size of practice location and federal state. We used Likert scales, multiple choice and open text formats. Open text answers regarding barriers of POCT use were categorized into categories and subcategories in a joint deductive-inductive approach by two researchers (AM, RM). The questionnaire included a short cover letter with information on the survey. The questionnaire was designed with the expertise of an interdisciplinary research team consisting of four experienced GPs and healthcare researchers (JB, FW, GS, IG), a work and organizational psychologist (AM) and a biochemist with expertise in primary care laboratory testing (RM). Following a participatory research approach, we discussed objectives, item selection, phrasing and feasibility of the questionnaire with GPs and other researchers within a 90 min research meeting in January 2021. During the design of the questionnaire, several GPs tested the pre-final questionnaire and commented on comprehensibility, feasibility and the scope of the included questions. Only anonymous data were collected and participants were informed in a written statement that returning the questionnaire to our institute implied their consent for anonymous participation in the study. The German questionnaire and an English translation are provided in the Additional file.

### Data analysis

Raw data from the questionnaire were entered into Microsoft Excel 2016. Statistical analyses were performed using R (version 4.1.2) [12] and RStudio (version 2021.09.2) [13]. Missing or invalid values were excluded from the analysis. The Pearson’s Chi^2^ test was used to test for statistically significant differences in categorical variables between groups. The Fisher's exact test for count data was performed when the Chi^2^ approximation was inadequate, e.g. when sample sizes were small or the data were very unequally distributed among the cells of the table. The Kruskal–Wallis rank sum test was used to compare continuous variables (i.e. number of utilized POCTs per GP) from more than two independent groups of equal or different sample sizes. Pearson's product-moment correlation was used to analyse any potential association between the mean number of utilized POCTs per GP and (i) the work experience as GP and (ii) time to receive test results from external laboratories.

## Results

### Characteristics of the surveyed general practitioners

From the 2,014 GPs reached, 292 responded and participated in our study, resulting in a response rate of 14.5%. The characteristics of the participating GPs are summarized in Table 1. The majority of participating GPs (75%) had 8 years or more work experience as a GP (median: 15 years, interquartile range: 8—23 years) and 59% were female. Most surveyed GPs were self-employed (85%) and worked in either single-handed (58%) or group practices (33%). The population size of the practice location was nearly equally distributed between rural communities (< 5,000 pop.), small towns (5,000—20,000 pop.), large towns (20,000—100,000 pop.) and urban centres (> 100,000 pop.).

**Table 1:** Characteristics of general practitioners (GPs) surveyed

**Number of GPs reached**	2,014	
**Number of GPs returning questionnaire (n, %**^**a**^**)**	292	Response rate: 14.5%
**Gender (n, %**^**a**^**)**		
Female	170	59.4%
Male	116	40.6%
NR	6	-
**Work experience as GP (median, interquartile range)**	15 years	IQR: 8 – 23
**Employment status (n, %**^**a**^**)**		
Self-employed GP	244	85.0%
Employed in general practice	43	15.0%
NR	5	-
**Practice type (n, %**^**a**^**, 95% CI)**		
Single-handed practice	168	57.9%
Group practice	95	32.8%
Ambulatory healthcare centre	25	8.6%
Other	2	0.7%
NR	2	-
**Population size of practice location (n, %**^**a**^**)**		
Rural community (< 5,000 pop.)	84	29.3%
Small town (5,000—20,000 pop.)	70	24.5%
Large town (20,000—100,000 pop.)	65	22.7%
Urban centres (> 100,000 pop.)	67	23.4%
NR	6	-
**German federal state (n, %**^**a**^**)**		
Thuringia	212	73.1%
Bremen	45	15.5%
Bavaria [Lower Franconia]	31	10.7%
Other	2	0.7%
NR	2	-

*NR* Not reported or invalid answer

^a^Percentage among valid answers (excluding NR)

### Utilization of POCTs among general practitioners

**Fig. 1:**
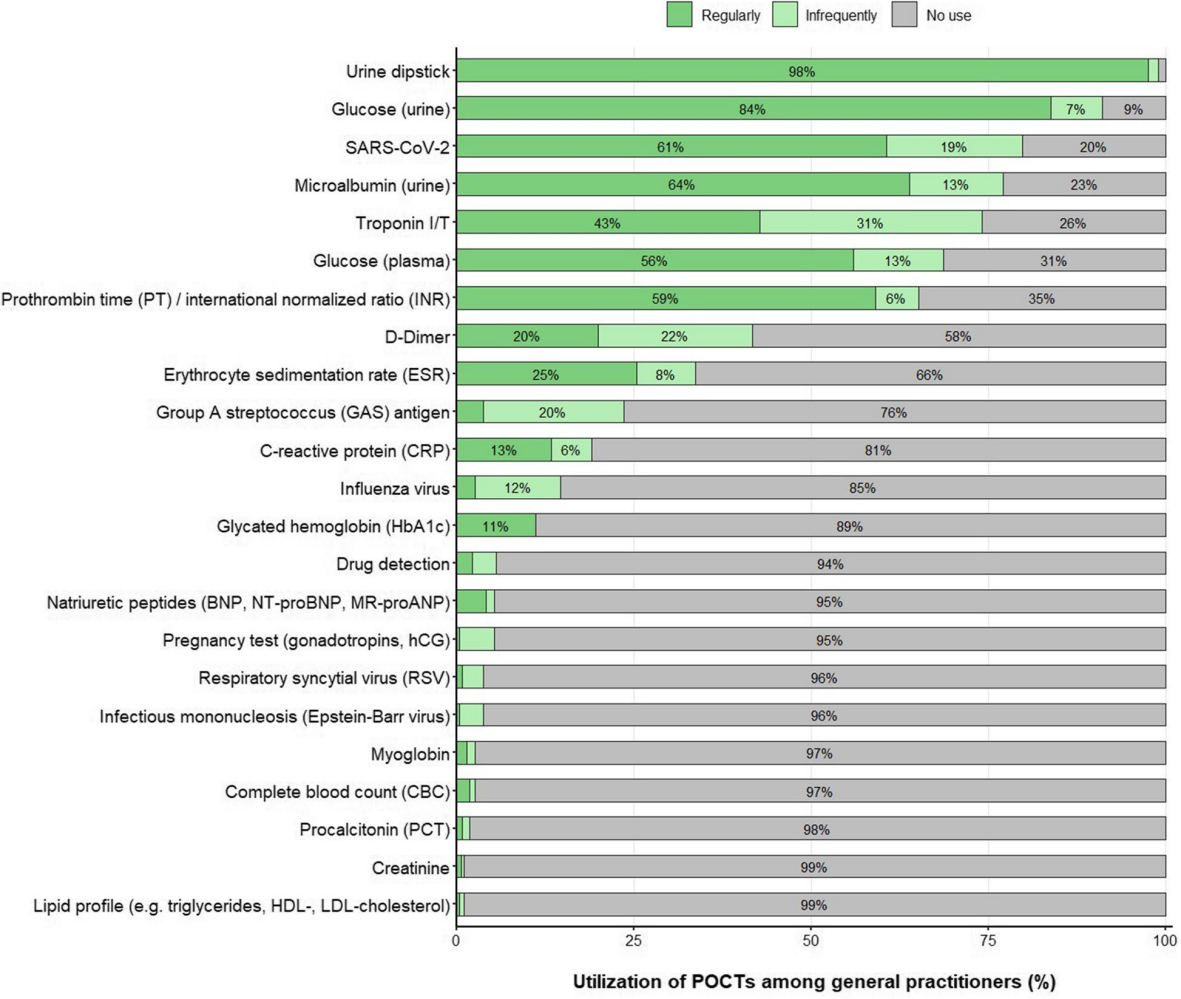
Utilization of POCTs among general practitioners. Utilization of POCTs reported by general practitioners (GPs) in Germany as percentages among all surveyed GPs (*n* = 292). Dark green: regular use (≥ 1 × in 14 days), light green: infrequent use (< 1 × in 14 days), grey: no use. Percentages within the bars are displayed if > 5%

Our survey shows that six POCTs are used by the majority of surveyed GPs (> 50%), which include urine dipstick tests (99%), glucose (urine [91%] and plasma [69%]), SARS-CoV-2 (80%), urine microalbumin (77%), troponin I/T (74%) and prothrombin time / international normalized ratio (65%) (Fig. 1). POCTs for D-dimer, erythrocyte sedimentation rate (ESR), Group A streptococcus (GAS), C-reactive protein (CRP), influenza virus and glycohemoglobin (HbA1c) are used by much fewer GPs in Germany (11- 42%). Other POCTs, such as natriuretic peptides or procalcitonin (PCT) are utilized only by very few GPs (< 6%). Participating GPs rarely reported the use of other POCTs than those included in our questionnaire: vitamin D (*n* = 2), SARS-CoV-2 antibodies (*n* = 1), uric acid (*n* = 1), thyroid-stimulating hormone (TSH) (*n* = 1), *Heliobacter pylori* (*n* = 1) and *Borrelia* antibodies (*n* = 1), all of them available as POCTs for professional use in GP practices. Our analyses do not support that GPs from rural areas more frequently utilize troponin I/T POCTs compared to their colleagues from towns or urban centres (*p* = 0.056, see Additional file sTable 1 for detailed test statistics).

The median number of POCTs used per GP (either regularly or infrequently) was 7.0 (IQR: 5.0–8.0) and the median for regularly utilized POCTs was 5.0 (IQR: 4.0–7.0). The number of utilized POCTs (either regularly or infrequently) did not differ between GP practice type (i.e. single-handed, group practice and ambulatory healthcare centre; *p* = 0.307) and population size of GP practice location (i.e. rural community, towns and urban centres; *p* = 0.099) (see Additional file sTable 2). Similarly, work experience as GP and the time to receive test results from external laboratories were not associated with the number of utilized POCTs (*p* = 0.866 and *p* = 0.139, respectively).

### Perceived usefulness of POCTs in different primary care settings and barriers of POCT use in GP practices

**Fig. 2:**
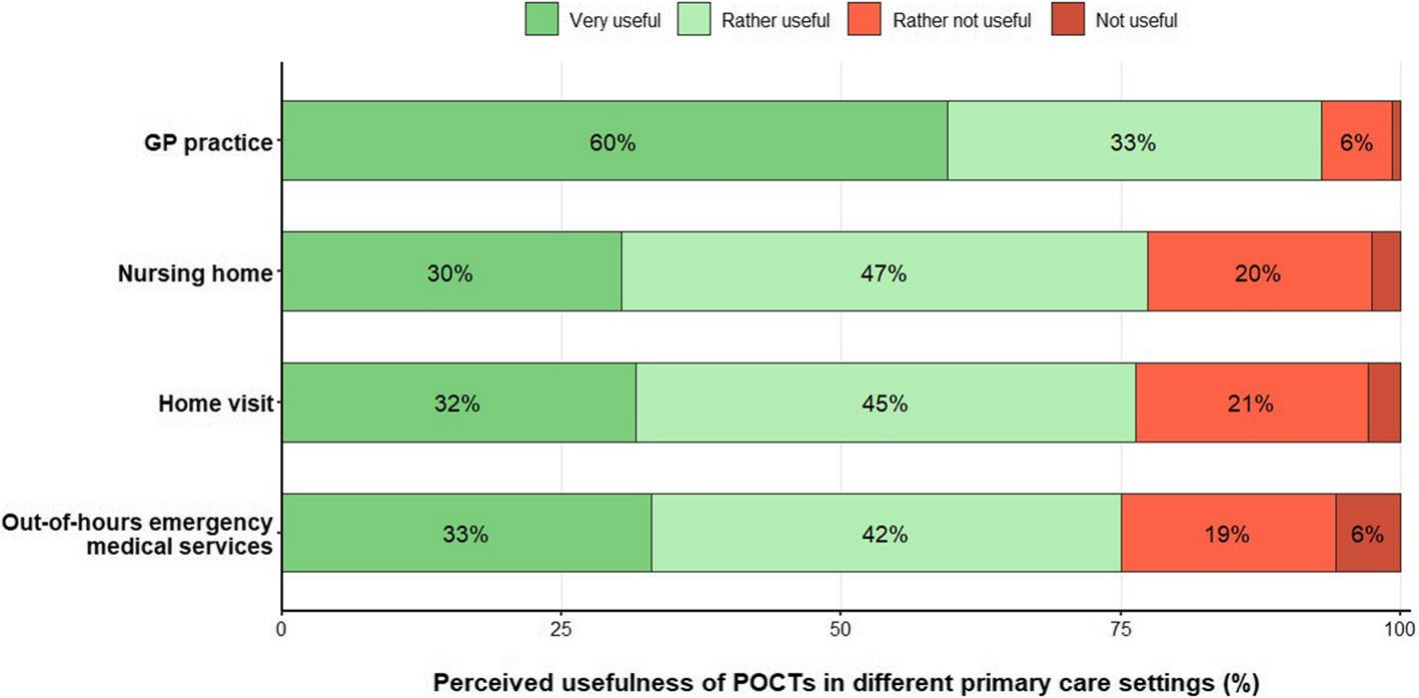
Perceived usefulness of POCTs in different primary care settings. Reported by general practitioners (GPs) in Germany as percentages among all surveyed GPs (*n* = 292); percentages within the bars are displayed if > 5%

Nearly all (93%) of the participating GPs perceived POCTs as very or rather useful diagnostic tools in the GP practice, while 7% rated POCTs as not useful (Fig. 2). The reported usefulness of POCTs in the GP practice was not different between GP practice type and population size of the practice location (*p* = 0.825 and *p* = 0.533, respectively, see Additional material sTable 3). Although the majority (> 75%) of the surveyed GP perceived POCTs as useful in other primary care settings (i.e., home visits, medical services in nursing homes, and out-of-hours emergency medical services), about 25% of the GPs rated POCT use as not useful in these settings. When asked for a prediction on the importance of POCTs in the next ten years compared to today, the majority (65%) of the surveyed GPs predicted that the importance of POCTs will increase, while 30% of GPs predicted that the importance of POCTs will not change. Relatively few GPs (5%) think that POCTs will lose relevance in the next ten years.

When asked for major limitations and barriers of POCT use in their GP practice, 231 GPs (79%) answered this question. Answers were provided in an open text format and were categorized into five categories. The most frequently (*n* = 155) reported limitation of POCT use were economic aspects, including high costs of POCTs and inadequate reimbursement (Table 2). Test-related limitations (*n* = 91), especially concerns regarding diagnostic accuracy, and difficulties to integrate POCT-testing into practice routines (e.g. time and personnel expenses) were also frequently (*n* = 81) mentioned by participating German GPs as limitations for POCT use. Clinical aspects, such as low frequency of test occasion in GP practices or low impact on clinical decisions, were mentioned relatively infrequently (*n* = 18) by GPs.

**Table 2:** Perceived limitations and barriers of POCT use in GP practices

Category and subcategory	Example
**Economic aspects (** ***n*** ** = 155)**	
High costs (*n* = 105)	“Costs for devices and test stripes”
Inadequate or lack of reimbursement (*n* = 50)	“Reimbursement does not cover the costs or lack of reimbursement”
**Test-related aspects (** ***n*** ** = 92)**	
Inferior diagnostic accuracy (*n* = 56)	“Poor sensitivity/specificity”
Limited shelf life (*n* = 17)	“[Limited] shelf life, due to infrequent test use”
Difficult handling (*n* = 8)	“Insecurity of medical staff in performing the test”
Required maintenance of devices (*n* = 6)	“Frequent maintenance procedures of the devices”
Other (*n* = 5)	“Sampling material (pre analytical steps)”
**Integrability into practice routine (** ***n*** ** = 81)**	
Time expenses (*n* = 33)	“Time disadvantages, e.g. time to result; [POCTs] often not feasible in crowded GP practice”
Personnel expenses (*n* = 14)	“Substantial personnel expenses”
Organizational consequences (*n* = 14)	“Integrability into daily practice routines”
Purchase of POCT devices (*n* = 11)	“Various suppliers, confusing”
Space requirement, incl. storage space (*n* = 5)	“Laboratory [space] required”; “Storage of test equipment”
Other (*n* = 4)	“Bureaucratic burden”
**Clinical aspects (** ***n*** ** = 18)**	
Limited clinical value (*n* = 11)	“Not all tests are useful in rural GP practice”; “Often, they [POCTs] do not help me in my decision”
Low frequency of test occasion (*n* = 7)	“Infrequent use”; “Low utilization rate of devices”
**Other (** ***n*** ** = 6)**	
	“Clinical experiences of GPs very heterogeneous”; “Fear of regress claims”

Participating GPs (*n* = 292) were asked to report barriers and limitations of POCT utilization in GP practice (open text format). 231 GPs (79%) answered this question and mentioned a total 352 limitations and barriers. Answers were categorized into categories and subcategories in a joint deductive-inductive approach

### GPs’ requirements for POCTs

In order to be feasible for routine use, a maximum test duration (from sampling to test result) of 10 min is accepted by 25% of GPs, while a duration of more than 20 min is only adequate for 25% of GPs (median: 15, IQR: 10–20).

When asked on the importance of relevant POCT characteristics, participating GPs ranked high diagnostic accuracy, high user-friendliness (including being integrable into practice routines) and fast test results as the most important characteristics (Table 3). Quantitative test result output and appropriate shelf life were ranked as the least important POCT properties.

**Table 3:**
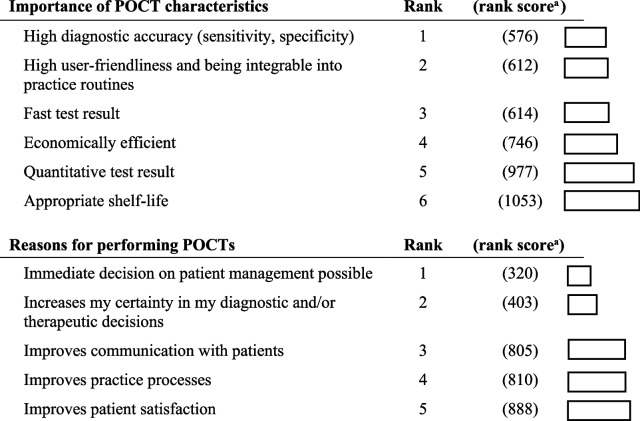
Ranking of relevant POCT characteristics and reasons for performing POCTs

^a^GPs were asked to provide a ranking of predefined (i) POCT properties and (ii) reasons for performing POCTs by sorting from most important (1) to least important ((i) 6, (ii) 5). In order to calculate a summarised score for each POCT property, the scores from all questionnaires were added up. An analogue calculation was conducted for the ranking of reasons for performing POCTs

Regarding the reason for performing POCTs, GPs ranked immediate decisions on patient management and the increase of diagnostic certainty as the most important reasons (Table 3). Improved patient satisfaction was ranked as the least important reason for performing POCTs.

## Discussion

In this study, we surveyed 292 German GPs on their utilization, perspectives and requirements on point-of-care testing in primary care. Our study shows that the participating GPs utilize only six POCTs on average in their routine work. However, the great majority of GPs rate POCTs as useful diagnostic tools in the GP practice and in other primary care settings, such as home visits and out-of-hours emergency medical services. Most surveyed GPs predict that POCTs will gain importance over the next ten years. On the other hand, GPs reported several limitations and barriers for POCT use, most noticeable economic aspects (i.e. high costs and lack of adequate reimbursement) and concerns regarding diagnostic test performance as well as time and personnel expenses.

### POCT utilization

Our results on the most frequently utilized POCTs are similar to a survey conducted among GPs in the German federal state of Saxony in early 2020 [7]. With the exception of SARS-CoV-2 rapid tests, all frequently utilized POCTs are available for in-vitro-diagnostic purposes since many years. Given the importance of fast test results during the ongoing COVID-19 pandemic, German GPs quickly implemented SARS-CoV-2 POCTs, including molecular tests that are now widely accepted among GPs, medical staff and patients [14, 15]. Notably, besides SARS-CoV-2 rapid tests, only relatively few German GPs surveyed in our study utilize POCTs for infectious diseases, such as CRP (utilized by less than 20% of GPs), influenza (15%), RSV (< 5%) and procalcitonin (< 5%). In contrast, GPs in other European countries, such as Norway and Sweden, more frequently use POCTs (e.g., CRP, group A streptococcus [> 65%]) for acute infections [16].

### Factors related to POCT utilization in GP practices

Our survey shows that the great majority of German GPs rate POCTs as useful diagnostic tools in different primary care settings. The main reasons for performing POCTs are the possibility to decide immediately on further patient care as well as to increase diagnostic certainty of GPs. The positive attitude of participating German GPs towards POCT use is somewhat contrary to the relatively limited spectrum of POCTs actually used in practice, which is explained by multiple factors, including factors on the level of the individual GP but also contextual factors related to the German healthcare system.

A frequently reported limitation by the surveyed GPs is the perceived low diagnostic test performance of POCTs. Although GPs often express concerns regarding test performance, the diagnostic accuracy of many POCTs have improved over time and many are not inferior to standard laboratory tests [17]. Moreover, GPs frequently reported difficulties in integrating POCT-testing into practice routines, which also includes time expenses. In German GP practices, the patient consultation time is 7 min on average [18], which leaves little time for additional diagnostics measures, including POCTs. Importantly, in many treatment situations, rather than using POCTs, GPs base their clinical decision on their clinical experience, patient symptoms and anamnesis as well as external central laboratory analyses.

In addition to these factors, POCT use in GP practices is influenced by factors related to the German healthcare system, such as reimbursement regulations. In fact, lack of adequate reimbursement and high costs of POCTs are the most reported barriers of POCT use. In Germany, reimbursement of in-vitro laboratory tests in GP practices often does not exceed 1.50€ for patients with statutory health insurance (approx. 88% of the German population). As a result, performing POCTs is often not economical efficient for a GP. Moreover, Germany has a sufficient infrastructure of external central laboratories that often provide results on the same day or early the next day after sample collection, which may reduce the actual need for performing POCTs in GP practices. In our survey, 75% of the GPs reported that results from external laboratory analyses are usually available after less than 18 h when the patient sample was sent to the laboratory before noon.

In addition, a variety of other factors is relevant for the GPs’ decision to implement a POCT in practice, but evaluation studies in primary care do often not address these aspects [19]. For example, our survey shows that high user-friendliness and being integrable into practice routines are major test characteristics that POCTs should possess to be considered for use in GP practices. In addition, the views of patients may also play a significant role in German GPs’ consideration of POCTs, which should be addressed in future studies.

### GP characteristics and POCT utilization

There is evidence that GPs in rural areas more commonly apply certain POCTs compared to their colleagues in urban areas, such as troponin I/T [20]. However, our analyses show that troponin I/T use as well as overall POCT utilization and perceived usefulness were not different between GPs from rural areas and those from urban centers. This might be explained by the fact that the ambulatory healthcare infrastructure (laboratory infrastructure [21], availability of GP practices and hospitals [22], etc.) is relatively homogenous across Germany. Moreover, in our study, POCT utilization and perceived usefulness was independent from the clinical experience and practice type. Similarly, a survey among primary care physicians from UK could not identify any significant relationships between demographic factors (time to receive blood test results, distance of practice from nearest emergency department and practice size) and the numbers of conditions for which respondents considered that POCTs would be helpful [23].

### Representativeness of the surveyed GPs

The relatively low participation rate of 14.5% and inclusion of GPs from only three out of sixteen German federal states in combination with the overrepresentation of Thuringian GPs raises the question on the generalizability of our results. However, when compared to official statistics [22, 24], our sample of 292 GPs is reasonably representative for the German GP population. For example, the distribution of different practice types as well as the rate of self-employed GPs is very similar to the total German GP population. Moreover, the population size of practice locations resembles the distribution of urban and rural areas in Germany, although in our study GPs from rural areas are slightly overrepresented. Similarly, female GPs are overrepresented (60%) in our study (national ratio of female GPs: 49%).

### Strengths and limitations

From our knowledge, our study is the first survey among German GPs on POCT utilization, limitations and attitudes towards POCT diagnostics. The questionnaire was designed by an interdisciplinary research team consisting of GPs and researchers in the field of POCT application. We cannot exclude a selection bias towards GPs with more positive attitude towards POCTs since participation was voluntary. Another limitation is that many questions asked for overall views on POCT use, although answers often dependent on the specific POCT, clinical situation and contextual factors. We addressed this limitation during the design of the questionnaire by selecting aspects and choosing a wording that allowed an overall rating. In addition, participants were asked in the introductory text to take on a superordinate role and give answers that were as generally valid as possible.

## Conclusion

German GPs rate POCTs as useful diagnostic tools as they support clinical decision-making immediately at the point-of-care and increase their diagnostic certainty. However, several barriers result in a relatively low utilization of POCTs, including high costs, perceived inferior diagnostic accuracy as well as increased time and personnel expenses. Moreover, contextual factors also contribute to the slow implementation of POCTs in German GP practices, such as inadequate reimbursement, sufficient central laboratory infrastructure as well as scarce evidence on POCT effectiveness in the primary care setting. Further research is needed to assess the value of POCTs, addressing aspects that are relevant for GPs, such as the impact on clinical decision-making and practice routines as well as user-friendliness and clinical effectiveness.

## Supplementary Information

**Additional file 1: Questionnaire (English translation). sTable 1** Use of troponin by population size of GP practice location. **sTable 2. **Number of utilized (either regularly or infrequently) POCTs per GP by various GP characteristics. **sTable 3. **Perceived usefulness of POCTs in GP practice by various GP characteristics.  

## Data Availability

The datasets used and/or analysed during the current study are available from the corresponding author on reasonable request.

## Abbreviations

COVID-19: Coronavirus disease 2019
CRP: C reactive protein
GP: General practitioner
POCT: Pointof-care-test
RSV: Respiratory syncytial virus
SARS-CoV-2: Severe acute respiratory syndrome coronavirus 2

## References

[1] Junker R, Schlebusch H, Luppa PB (2010). Point-of-care testing in hospitals and primary care. Deutsches Arzteblatt international.

[2] Schols AMR, Dinant G-J, Hopstaken R, Price CP, Kusters R, Cals JWL. International definition of a point-of-care test in family practice: a modified e-Delphi procedure. Fam Pract. 2018;35(1):4–12. 10.1093/fampra/cmx134 29385437

[3] Luppa PB, Müller C, Schlichtiger A, Schlebusch H (2011). Point-of-care testing (POCT): Current techniques and future perspectives. Trends Analyt Chem.

[4] St John A, Price CP (2014). Existing and Emerging Technologies for Point-of-Care Testing. Clin Biochem Rev.

[5] Matthes A, Bleidorn J, Markwart R. Research on point-of-care tests in outpatient care in Germany: A scoping review and definition of relevant endpoints in evaluation studies. Z Evid Fortbild Qual Gesundhwes. 2022;174:1–10. 10.1016/j.zefq.2022.06.002 36055890

[6] Verbakel JY, Turner PJ, Thompson MJ, Plüddemann A, Price CP, Shinkins B (2017). Common evidence gaps in point-of-care diagnostic test evaluation: a review of horizon scan reports. BMJ Open.

[7] Oehme R, Sandholzer-Yilmaz AS, Heise M, Frese T, Fankhaenel T (2022). Utilization of point-of-care tests among general practitioners, a cross-sectional study. BMC Prim Care.

[8] Kip MMA, Hummel JM, Eppink EB, Koffijberg H, Hopstaken RM, IJzerman MJ, et al. Understanding the adoption and use of point-of-care tests in Dutch general practices using multi-criteria decision analysis. BMC Fam Pract. 2019;20(1):8. 10.1186/s12875-018-0893-4 PMC6327588 30630430

[9] Howick J, Cals JW, Jones C, Price CP, Plüddemann A, Heneghan C (2014). Current and future use of point-of-care tests in primary care: an international survey in Australia, Belgium, The Netherlands, the UK and the USA. BMJ Open.

[10] Jones CH, Howick J, Roberts NW, Price CP, Heneghan C, Plüddemann A (2013). Primary care clinicians' attitudes towards point-of-care blood testing: a systematic review of qualitative studies. BMC Fam Pract.

[11] Edwards P, Roberts I, Clarke M, DiGuiseppi C, Pratap S, Wentz R, et al. Methods to increase response rates to postal questionnaires. Cochrane Database Syst Rev. 2007;(2):MR000008. 10.1002/14651858.MR000008.pub3 17443629

[12] R Core Team. R: A language and environment for statistical computing. Vienna, Austria: R Foundation for Statistical Computing; 2022. URL https://www.R-project.org/.

[13] RStudio Team. RStudio: Integrated Development for R Boston, MA, USA2020 [Available from: http://www.rstudio.com/.

[14] Matthes A, Wolf F, Bleidorn J, Markwart R (2022). "It Was Very Comforting to Find Out Right Away." - Patient Perspectives on Point-of-Care Molecular SARS-CoV-2 Testing in Primary Care. Patient Prefer Adherence.

[15] Wolf F, Matthes A, Markwart R, Bleidorn J. Perspectives of physicians and medical assistants on the implementation of NAAT-based point-of-care testing for SARS-CoV-2 in primary care in Germany. Z Evid Fortbild Qual Gesundhwes. 2022;175:43–9. 10.1016/j.zefq.2022.09.006 PMC9648907 36372644

[16] van der Velden AW, van de Pol AC, Bongard E, Cianci D, Aabenhus R, Balan A, et al. Point-of-care testing, antibiotic prescribing, and prescribing confidence for respiratory tract infections in primary care: a prospective audit in 18 European countries. BJGP Open. 2022;(2):BJGPO.2021.0212. 10.3399/BJGPO.2021.0212 PMC9447323 34920989

[17] Howick J, Bossuyt PM, Cals JWL (2017). Point of care testing in family practice: common myths debunked. Fam Pract.

[18] Irving G, Neves AL, Dambha-Miller H, Oishi A, Tagashira H, Verho A (2017). International variations in primary care physician consultation time: a systematic review of 67 countries. BMJ Open.

[19] Lingervelder D, Koffijberg H, Kusters R, IJzerman MJ (2019). Point-of-care testing in primary care: A systematic review on implementation aspects addressed in test evaluations. Int J Clin Pract.

[20] Blank WA, Schmidt R, Schneider A (2009). Use of troponin T testing in familiy practice: Use and benefit of troponin T testing based on a census of Bavarian family doctors. Z Allgmed.

[21] Höpfner T, Militzer-Horstmann, C., Schäffer, T., Schmiedel, L., Schrey, C., Kurscheid, C. & Mollenhauer, J. Studie zur Identifikation von Zusammenhängen zwischen der Trägerschaft und der Qualität labormedizinischer Leistungserbringung in Deutschland. Studie im Auftrag des ALM e. V. – Akkreditierte Labore der Medizin e. V. Leipzig, Germany: Akkreditierte Labore der Medizin e. V.; 2021.

[22] Kassenärztliche Bundesvereinigung. Gesundheitsdaten Berlin: Kassenärztliche Bundesvereinigung; 2022 [Available from: https://gesundheitsdaten.kbv.de/cms/html/17019.php.

[23] Turner PJ, Van den Bruel A, Jones CH, Plüddemann A, Heneghan C, Thompson MJ (2016). Point-of-care testing in UK primary care: a survey to establish clinical needs. Fam Pract.

[24] Bundesärztekammer. Ärztestatistik zum 31. Dezember 2021. In: Bundesärztekammer, editor. Berlin, Germany: Bundesärztekammer; 2022. p. 48.

