# Supplementary material for: Point-of-care laboratory testing in primary care: utilization, limitations and perspectives of general practitioners in Germany
Source: BMC Prim Care. 2023 Apr 11;24:96. doi: 10.1186/s12875-023-02054-0 (PMC10088261; doi:10.1186/s12875-023-02054-0)
Supplement: Supplementary file 1 — Additional file 1: Questionnaire (English translation). sTable 1 Use of troponin by population size of GP practice location. sTable 2. Number of utilized (either regularly or infrequently) POCTs per GP by various GP characteristics. sTable 3. Perceived usefulness of POCTs in GP practice by various GP characteristics. [file 12875_2023_2054_MOESM1_ESM.docx]

**Additional File**

**Point-of-Care laboratory testing in primary care: Utilization, limitations and perspectives of general practitioners in Germany**

Anni Matthes^1, 2^, Florian Wolf^1^, Guido Schmiemann^3^, Ildikó Gágyor^4^, Jutta Bleidorn^1^, Robby Markwart^1, 2^

^1^ Institute of General Practice and Family Medicine, Jena University Hospital, Friedrich Schiller University, Jena, Germany

^2^ InfectoGnostics Research Campus Jena, Jena, Germany

^3^ Institute for Public Health and Nursing Sciences, Department for Health Services Research, Bremen University, Bremen, Germany

^4^ Department of General Practice, University Hospital Wuerzburg, Wuerzburg, Germany.

**Correspondence**: robby.markwart@med.uni-jena.de

Jena University Hospital, Institute of General Practice and Family Medicine, Bachstr. 18, 07743 Jena, Germany

Tel +49 3641 939 5820

Email: robby.markwart@med.uni-jena.de

**Table of Contents**

[Questionnaire (English translation) 3](#_Toc130814744)

[Use of troponin by population size of GP practice location 7](#_Toc130814745)

[Number of utilized POCTs per GP by various GP characteristics 7](#_Toc130814746)

[Perceived usefulness of POCTs in GP practice by various GP characteristics 8](#_Toc130814747)

# Questionnaire (English translation)

**Questionnaire: Utilization of POCTs in the GP practice**

| **How often do you use POCTs for the following laboratory analyses in your routine work as a GP?**  Please mark. | | | **Regularly as POCT** (≥ 1x in 14 days) | **Infrequently as POCT**  (< 1x in 14 days) | **No use as POCT** |
| --- | --- | --- | --- | --- | --- |
| **Blood analyses** | | | | | |
| A01 | ESR (erythrocyte sedimentation rate) | | 🞏_1_ | 🞏_2_ | 🞏_3_ |
| A02 | CRP (C-reactive protein) | | 🞏_1_ | 🞏_2_ | 🞏_3_ |
| A03 | PCT (procalcitonin) | | 🞏_1_ | 🞏_2_ | 🞏_3_ |
| A04 | Complete blood count (erythrocytes, leukocytes, thrombocytes; haemoglobin, haematocrit) | | 🞏_1_ | 🞏_2_ | 🞏_3_ |
| A05 | Lipid profile (e.g. triglycerides, HDL-, LDL-cholesterol) | | 🞏_1_ | 🞏_2_ | 🞏_3_ |
| A06 | Natriuretic peptides (BNP, NT-proBNP, MR-proANP) | | 🞏_1_ | 🞏_2_ | 🞏_3_ |
| A07 | Myoglobin | | 🞏_1_ | 🞏_2_ | 🞏_3_ |
| A08 | Troponin I, troponin T | | 🞏_1_ | 🞏_2_ | 🞏_3_ |
| A09 | D-Dimer | | 🞏_1_ | 🞏_2_ | 🞏_3_ |
| A10 | Prothrombin time (PT) / international normalized ratio (INR) | | 🞏_1_ | 🞏_2_ | 🞏_3_ |
| A11 | Plasma glucose | | 🞏_1_ | 🞏_2_ | 🞏_3_ |
| A12 | Glycated hemoglobin (HbA1c) | | 🞏_1_ | 🞏_2_ | 🞏_3_ |
| A13 | Creatinine | | 🞏_1_ | 🞏_2_ | 🞏_3_ |
| A14 | Pregnancy test (gonadotropins, hCG) | | 🞏_1_ | 🞏_2_ | 🞏_3_ |
| **Urine analyses** | | | | | |
| A15 | Urine dipstick (protein, erythrocytes, leucocytes, nitrite, etc.) | | 🞏_1_ | 🞏_2_ | 🞏_3_ |
| A16 | Glucose | | 🞏_1_ | 🞏_2_ | 🞏_3_ |
| A17 | Microalbumin | | 🞏_1_ | 🞏_2_ | 🞏_3_ |
| **Nasal and throat swab** | | | | | |
| A18 | Infectious mononucleosis (Epstein-Barr virus) | | 🞏_1_ | 🞏_2_ | 🞏_3_ |
| A19 | Group A streptococcus (GAS) antigen | | 🞏_1_ | 🞏_2_ | 🞏_3_ |
| A20 | Influenza viruses | | 🞏_1_ | 🞏_2_ | 🞏_3_ |
| A21 | RSV (respiratory syncytial virus) | | 🞏_1_ | 🞏_2_ | 🞏_3_ |
| A22 | SARS-CoV-2 | | 🞏_1_ | 🞏_2_ | 🞏_3_ |
| **Other analyses** | | | | | |
| A23 | Drug detection | | 🞏_1_ | 🞏_2_ | 🞏_3_ |
| **Other analyses:** | | | | | |
| A24 | _____________________________________________________________________________ _____________________________________________________________________________ | | | | |
| B01 | **For which clinical situations or laboratory analysis would you like to use a POCT?** | _________________________________________ _________________________________________ _________________________________________ | | | |

| C01 | **If you take a blood sample before noon, how long does it usually take to receive the test result from the external laboratory?** | hours  to |
| --- | --- | --- |

| **How do you rate the value of POCTs in the following primary care settings?**  Please mark! | | **Very useful** | **Rather useful** | **Rather not useful** | **Not useful** | **Don’t know/**  **Prefer not to say** |
| --- | --- | --- | --- | --- | --- | --- |
| D01 | GP practice | 🞏_1_ | 🞏_2_ | 🞏_3_ | 🞏_4_ | ⭘_5_ |
| D02 | Home visits | 🞏_1_ | 🞏_2_ | 🞏_3_ | 🞏_4_ | ⭘_5_ |
| D03 | Nursing homes | 🞏_1_ | 🞏_2_ | 🞏_3_ | 🞏_4_ | ⭘_5_ |
| D04 | Out-of-hours emergency medical services | 🞏_1_ | 🞏_2_ | 🞏_3_ | 🞏_4_ | ⭘_5_ |

| E01 | **What are the most important limitations/barriers of POCT use in your practice?** | _________________________________________ _________________________________________ _________________________________________ |
| --- | --- | --- |

| F01 | **In our opinion: Compared to today, in the next 10 years POCTs will** | 🞏_1_  gain importance  🞏_2_  have the same importance  🞏_3_  lose importance |
| --- | --- | --- |
| F02 | **Please give reasons for your answer:** | _________________________________________ _________________________________________ |

| **Please create a ranking of the following properties of POCTs. Please provide scores for each property from 1 (very important) to 6 (less important).**  **Award each score only once.** |  | **The POCT** |
| --- | --- | --- |
|  | G01 | has a sufficient shelf-life |
|  | G02 | is user-friendly and is integrable into practice routines |
|  | G03 | is economically efficient |
|  | G04 | provides fast test results |
|  | G05 | provides a quantitative test result |
|  | G06 | has a good diagnostic test performance (sensitivity, specificity) |

| **Below you find consequences that may result from performing a POCT in your GP practice.**  **Please create a ranking of these consequences and provide scores for each consequence from 1 (very important) to 5 (less important).**  **Award each score only once.** |  | **The use of POCTs** |
| --- | --- | --- |
|  | H01 | increases my certainty in my diagnostic and therapeutic decisions |
|  | H02 | improves communication with patients |
|  | H03 | makes immediate decisions on patient management possible |
|  | H04 | improves practice processes (e.g., patient flows and practice organisation) |
|  | H05 | improves patient satisfaction |

| I01 | **In order to be feasible in practice routine, what is the maximum time allowed for the performance of a POCT (from sampling to test result)?** | minutes |
| --- | --- | --- |

| **How open-minded towards technical innovations are…**  Please mark. | | **Very open** | **Rather open** | **Partly open** | **Rather not open** | **Not open** |
| --- | --- | --- | --- | --- | --- | --- |
| J01 | You? | 🞏_1_ | 🞏_2_ | 🞏_3_ | 🞏_4_ | 🞏_5_ |
| J02 | Other general practitioners? | 🞏_1_ | 🞏_2_ | 🞏_3_ | 🞏_4_ | 🞏_5_ |

| K01 | **If you could conduct research on POCT use in GP practices, what questions would you address?** | ____________________________________________ ____________________________________________ ____________________________________________ |
| --- | --- | --- |

| **We ask that you provide details about yourself and your practice.** | | |
| --- | --- | --- |
| L01 | Gender | 🞏_1_ Female 🞏_2_ Male 🞏_3_ Diverse |
| L02 | Working as a GP since |  |
| L03 | Specialisation | 🞏_1_ Working as a general practitioner  🞏_2_ Other, please specify _______________________________ |
| L04 | Employment status | 🞏_1_ Self-employed 🞏_2_ Employed 🞏_3_ Other |
| L05 | Practice type | 🞏_1_ Single handed practice 🞏_2_ Group practice 🞏_3_ Ambulatory healthcare centre 🞏_4_ Other, please specify_________________ |
| L06 | Population size of practice location | 🞏_1_ < 2.000 🞏_2_ 2.000 – 5.000 🞏_3_ > 5.000 – 20.000 🞏_4_ > 20.000 – 100.000 🞏_5_ > 100.000 |
| L07 | Federal State | 🞏_1_ Thuringia 🞏_2_ Bavaria 🞏_3_ Bremen 🞏_4_ Other, please specify _________________ |

| M01 | **Comments** |  |
| --- | --- | --- |

**Thank you for your participation!**

Please return the completed questionnaire to us in the enclosed return envelope.

# Use of troponin by population size of GP practice location

**sTable 1.** Use of troponin by population size of GP practice location

|  | **GPs from practices in rural communities**, n = 81 (n, %) | **GPs from practices in towns**, n = 130 (n, %) | **GPs from practices in urban** **centres**, n = 66 (n, %) |
| --- | --- | --- | --- |
| Regular use | 32 (39.0%) | 50 (38.5%) | 37 (56.1%) |
| Infrequent use | 29 (35.4% | 38 (29.2% | 19 (28.8% |
| No use | 21 (25.6%) | 42 (32.3%) | 10 (15.2%) |
| Pearson’s Chi^2^ test: Χ^2^ = 9.222, df = 4, p = 0.056  Rural community: population < 5000; towns: 5000 - 100,000; urban centres: >100,000 | | | |

# Number of utilized POCTs per GP by various GP characteristics

**sTable 2.** Number of utilized (either regularly or infrequently) POCTs per GP by various GP characteristics

|  | **Median, mean and interquartile range (IQR) of utilized POCTs per GP** |  |
| --- | --- | --- |
| ***GP practice type*** | |  |
| Single-handed (n = 168) | Median: 7.0, mean: 6.9, IQR: 5.0 - 8.0 | Kruskal-Wallis rank sum test:  Kruskal-Wallis Χ^2^ = 8.936, df = 3, p-value = 0.307 |
| Group practice (n = 95) | Median: 7.0, mean: 7.1, IQR: 6.0 - 8.0 |  |
| Ambulatory healthcare centre (n = 25) | Median: 6.0, mean: 6.8, IQR: 4.8 - 8.3 |  |
| Other (n = 2) | - |  |
| ***Population size of GP practice location*** | |  |
| Rural communities (< 5000 pop., n = 84) | Median: 7.0, mean: 7.6, IQR: 5.5 - 9.0 | Kruskal-Wallis rank sum test:  Kruskal-Wallis Χ^2^ = 4.564, df = 2, p-value = 0.099 |
| Towns (5000 - 100,000, n = 135) | Median: 6.0, mean: 6.8, IQR: 5.0 - 8.0 |  |
| Urban centres (> 100,000, n = 67) | Median: 7.0, mean: 6.6, IQR: 5.0 - 8.0 |  |
| ***Years of work experience as GP*** | | |
| Correlation coefficient: 0.010 (95% confidence interval: -0.107, 0.127); t = 0.169, df = 279, p-value = 0.866 (Pearson's product-moment correlation) | | |
| ***Mean time to receive test results from external laboratories when the sample was sent to the laboratory before noon*** | | |
| Correlation coefficient: 0.091 (95% confidence interval: -0.030, 0.209); t = 0.149, df = 265,  p-value = 0.139 (Pearson's product-moment correlation) | | |

# Perceived usefulness of POCTs in GP practice by various GP characteristics

**sTable 3.** Perceived usefulness of POCTs in GP practice by various GP characteristics

|  | **Perceived usefulness (n, [%])** |
| --- | --- |
| ***GP practice*** | |
| Single-handed | Very useful: 94 (56.6%)  Rather useful: 61 (36.7%)  Rather not useful: 10 (6.0%)  Not useful: 1 (0.6%) |
| Group practice | Very useful: 58 (62.4%)  Rather useful: 27 (29.0%)  Rather not useful: 7 (7.5%)  Not useful: 1 (1.1%) |
| Ambulatory healthcare centre | Very useful: 16 (66.7%)  Rather useful: 7 (29.2%)  Rather not useful: 1 (4.2%)  Not useful: 0 |
| Fisher's Exact Test for Count Data: p = 0.825 (two-tailed) | |
| ***Population size of GP practice location*** | |
| Rural communities (< 5000 pop.) | Very useful: 50 (61.0%)  Rather useful: 28 (34.1%)  Rather not useful: 3 (3.7%)  Not useful: 1 (1.2%) |
| Towns (5000 - 100,000 pop.) | Very useful: 74 (56.1%)  Rather useful: 48 (36.3%)  Rather not useful: 10 (7.6%)  Not useful: 0 |
| Urban centres (> 100,000 pop.) | Very useful: 42 (62.7%)  Rather useful: 19 (28.4%)  Rather not useful: 5 (7.5%)  Not useful: 1 (1.5%) |
| Fisher's Exact Test for Count Data: p = 0.533 (two-tailed) | |
